# Prevalence and Impact of Thyroid Dysfunction in Patients With Chronic Pulmonary Obstructive Pulmonary Disorder: A Systematic Review and Meta-Analysis

**DOI:** 10.7759/cureus.54968

**Published:** 2024-02-26

**Authors:** Divine Besong Arrey Agbor, Moulika Kari, Rachel Chandra Harika Chukka, Manisha Guntha, Aung K Zin, Sandipkumar S Chaudhari, Sai Kumar Kurva, Adil Amin

**Affiliations:** 1 Internal Medicine, Richmond University Medical Center, Staten Island, USA; 2 Medical School, Kamineni Institute of Medical Sciences, Hyderabad, IND; 3 Medicine and Surgery, Kamineni Institute of Medical Sciences, Hyderabad, IND; 4 Internal Medicine, Andhra Medical College, Visakhapatnam, IND; 5 Internal Medicine, University of Medicine, Mandalay, Mandalay, MMR; 6 Cardiothoracic Surgery, University of Alabama at Birmingham, Birmingham, USA; 7 Family Medicine, University of North Dakota School of Medicine and Health Sciences, Fargo, USA; 8 Internal Medicine, Rajiv Gandhi Institute of Medical Sciences, Adilabad, IND; 9 Cardiology, Pakistan Navy Ship Shifa, Karachi, PAK

**Keywords:** systematic review and meta-analysis, impact, prevalence, chronic obstructive pulmonary disorder, thyroid dysfunction

## Abstract

Thyroid gland dysfunction (TGD) has been increasingly recognized as a potential comorbidity in patients with chronic obstructive pulmonary disease (COPD). This study was designed to determine the prevalence of TGD in COPD patients. This systematic review and meta-analysis was conducted according to the guidelines of Preferred Reporting Items for Systematic Reviews and Meta-Analyses (PRISMA). To comprehensively identify relevant studies, a systematic search was conducted in major electronic databases, including PubMed, Embase, and the Cumulative Index to Nursing and Allied Health Literature (CINAHIL). The search was limited to English-language studies published after 31 December 2000. To determine the prevalence of TGD and assess the impacts, we compared forced vital capacity (FVC) (%), forced expiratory volume in one second (FEV1) (%), partial pressure of oxygen (PaO2) (mmHg), and partial pressure of carbon dioxide (PaCO2) (mmHg) between patients with and without TGD. A total of nine articles were included in this meta-analysis. The sample size of included studies ranged from 50 to 309. The pooled prevalence of TGD in patients with COPD was 45% (95% CI: 25% to 65%). The most common form of TGD was hypothyroidism. The study identified a lack of significant associations between TGD and COPD severity or various characteristics, highlighting the need for future prospective multi-center research, particularly with larger sample sizes to determine the clinical factors and biomarkers affecting the development of thyroid dysfunction in this population.

## Introduction and background

Chronic obstructive pulmonary disease (COPD) stands out as a major global contributor to morbidity and mortality [1]. The risk escalates significantly with age, reaching its zenith in individuals over 60 [1]. In addition, smokers constitute a substantial portion of COPD patients, with smoking being a primary risk factor for the development and progression of the disease [1]. Recognizing the systemic nature of chronic inflammation in COPD, it is acknowledged that although it primarily occurs in the lungs, inflammatory cytokines extend beyond, initiating inflammation in other organs [2]. COPD is now acknowledged to affect not only the lungs and airways but the entire body, leading to systemic manifestations, including various endocrine disorders involving the pituitary, thyroid, gonads, adrenals, and pancreas [3]. 

The systemic nature of COPD is intricately linked with endocrine disorders, with the thyroid being a key focus. Thyroid hormones play a critical role beyond respiratory drive, impacting overall metabolism. In COPD, systemic inflammation and disruptions in endocrine homeostasis contribute to thyroid gland dysfunction (TGD) [4-5]. Blood gas abnormalities further exacerbate this, leading to hypothyroidism, hyperthyroidism, and nonthyroidal illness syndrome. Understanding the broader metabolic implications of thyroid hormones illuminates the complexity of their interaction with COPD, highlighting the need for comprehensive management strategies [6]. Moreover, TGD in COPD patients not only affects respiratory function but also influences systemic metabolism, exacerbating the disease's complexity [5]. The evidence is growing that thyroid gland function may be disturbed in COPD patients [7]. Blood gas abnormalities, including hypoxemia and hypercapnia, are prevalent in COPD due to impaired gas exchange. These disturbances can disrupt thyroid function, leading to hypothyroidism, hyperthyroidism, and nonthyroidal illness syndrome [6].

Some studies indicate a higher prevalence of thyroid diseases among COPD patients, with a population-based study in Madrid revealing a prevalence of 14.21%, surpassing the expected standardized prevalence of chronic diseases at 11.06% [8]. The general occurrence of thyroid disorders is estimated at 14-20% among stable COPD patients and spikes to 70% during exacerbations [9]. Thyroid diseases occur more frequently among women than men with COPD, mirroring trends in the general population [10].

Despite a limited number of studies on the prevalence of TGD in COPD, certain characteristics of COPD patients may elevate their risk of developing hypothyroidism and hyperthyroidism [2]. The severity of airway obstruction in these patients is also linked to impaired thyroid gland function [11]. Consequently, this meta-analysis aims to meticulously analyze existing studies, elucidating the prevalence of TGD in COPD and evaluating its profound impacts on disease progression and patient outcomes.

## Review

Methodology

This systematic review and meta-analysis was conducted according to the guidelines of Preferred Reporting Items for Systematic Reviews and Meta-Analyses (PRISMA). The protocol of this meta-analysis is registered in PROSPERO (CRD42024510607).

Search Strategy

To comprehensively identify relevant studies, a systematic search was conducted in major electronic databases, including PubMed, Embase, and the Cumulative Index to Nursing and Allied Health Literature (CINAHL). The search strategy involved a combination of medical subject headings (MeSH) terms and keywords related to TGD and COPD. Additionally, a thorough exploration of grey literature and conference proceedings was undertaken to minimize potential publication bias. We also manually screened reference lists of additional studies to identify additional studies relevant to the study topic. The search was limited to studies published after 31 December 2000 and published in the English language. The search was conducted by two authors (MK and MG) and disagreements between them were resolved through discussion and consensus.

Study Selection

The inclusion criteria for studies encompassed observational research designs, specifically cross-sectional, cohort, and case-control studies reporting on the prevalence of TGD in individuals diagnosed with COPD. Studies assessing the impact of TGD on COPD outcomes, including disease severity and characteristics were also included. Case reports, reviews, and studies lacking sufficient data for analysis were excluded from this study. The eligibility of studies was independently assessed by two reviewers (RC and AZ), with any discrepancies resolved through discussion or consultation with a third reviewer (DA).

Data Extraction

We created a standardized data extraction form using Microsoft Excel to systematically gather pertinent information from the selected studies. This comprehensive form encompassed essential variables, including study characteristics such as author name, publication year, study design, study region, sample size, participant demographics, and prevalence of TGD. To assess the impacts, we compared forced vital capacity (FVC) (%), forced expiratory volume in one second (FEV1) (%), partial pressure of oxygen (PaO2) (mmHg), and partial pressure of carbon dioxide (PaCO2) (mmHg) between patients with and without thyroid disease. The extraction process was conducted independently by two reviewers (SS and SK), each responsible for extracting data from the included studies. Any disagreement between them was resolved through discussion.

Quality Assessment

The methodological quality of the included studies was assessed through a systematic evaluation using the Newcastle-Ottawa Scale (NOS), a widely recognized tool for appraising the quality of non-randomized studies in meta-analyses. This assessment considered various criteria, including study design, participant selection, comparability, and outcome ascertainment. The NOS assigned stars to each study based on these criteria, with a higher star count indicating higher methodological quality. The quality assessment was conducted independently by two reviewers, and any disparities in ratings were resolved through consensus or consultation with a third reviewer.

Statistical Analysis

Statistical analysis was performed using RevMan v. 5.4.1 (The Cochrane Collaboration, Copenhagen, Denmark) and STATA v. 17.0 (StataCorp LLC, College Station, TX). To compute the pooled prevalence of thyroid dysfunction, a random-effect model was used and proportion was calculated along with a 95% confidence interval (CI). To compare the characteristics and outcomes between patients with and without thyroid dysfunction, the odds ratio (OR) was calculated with 95% CI for categorical variables, and the mean difference (MD) was computed for continuous variables using a random-effect model. We used random-effect models to deal with heterogeneity among the study results potentially due to variation in study setting, sample size, and baseline characteristics. Heterogeneity among the study results was determined using the I-square value. An I-square value of >50% showed significant heterogeneity among the study results.

Results

**Figure 1:**
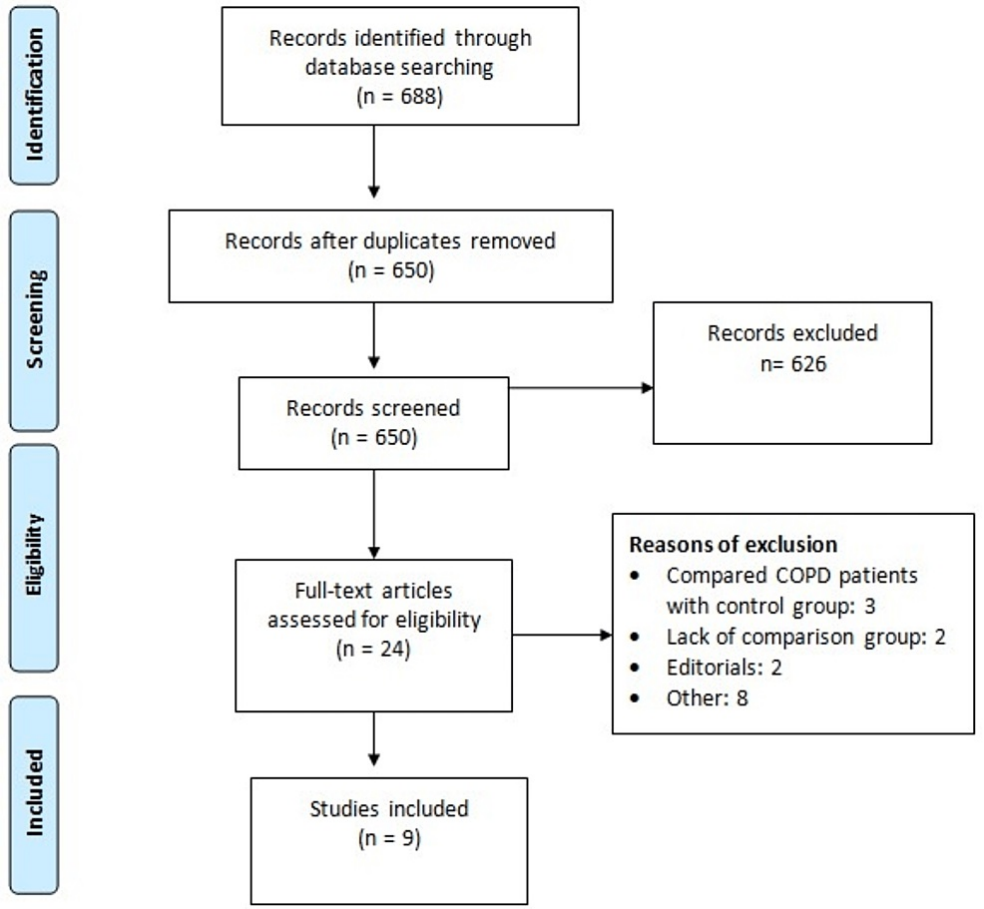
PRISMA flowchart of study selection

Online database searching identified 688 studies. Out of 688 studies, 38 were removed as duplicates. Through the initial screening, 626 studies were removed. Full-text of 24 articles was obtained and detailed assessment was done based on pre-defined inclusion and exclusion criteria. Finally, nine articles were included in this meta-analysis. Figure 1 shows the PRISMA flowchart of study selection. Out of these studies, four were conducted in India, three in Turkey, and one in China and Italy. The sample size of included studies ranged from 50 to 309. Table 1 shows the characteristics of the included studies. Table 2 shows the risk of bias assessment of the included studies.

**Table 1:** Characteristics of included studies

Author	Year	Study Design	Region	Sample Size	Thyroid Dysfunction (n)
Bahçecioğlu et al. [12]	2023	Cross-sectional	Turkey	78	53
Chaudhary et al. [6]	2018	Cross-sectional	India	171	43
Gumus et al. [13]	2021	Cross-sectional	Turkey	309	23
Huang et al. [14]	2021	Retrospective cohort	China	134	36
Sebasan et al. [15]	2021	Cross-sectional	India	50	32
Ulasli et al. [16]	2013	Case-control	Turkey	88	44
Terzano et al. [2]	2014	Cross-sectional	Italy	155	105
Singh et al. [17]	2016	Cross-sectional	India	201	130
Verma et al. [18]	2019	Cross-sectional	India	121	45

**Table 2:** Quality assessment of included studies

Author	Selection	Comparison	Assessment	Overall
Bahçecioğlu et al. [12]	2	2	2	Fair
Chaudhary et al. [6]	3	2	3	Good
Gumus et al. [13]	3	2	4	Good
Huang et al. [14]	3	2	3	Good
Sebasan et al. [15]	3	2	3	Good
Ulasli et al. [16]	2	2	3	Good
Terzano et al. [2]	3	2	2	Good
Singh et al. [17]	3	2	3	Good
Verma et al. [18]	2	1	2	Fair

Prevalence of Thyroid Dysfunction in COPD Patients

**Figure 2:**
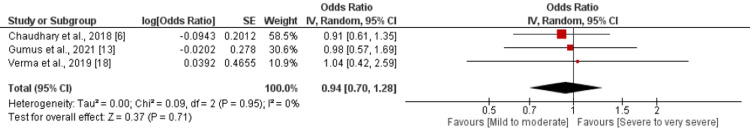
Association of thyroid dysfunction with Global Initiative for Chronic Obstructive Lung Disease stage (GOLD)

Overall, eight studies were included in the calculation of the pooled prevalence of thyroid dysfunction in COPD patients. The pooled prevalence of thyroid dysfunction in patients with COPD was 45% (n= 457) (95% CI: 25% to 65%). We assessed the prevalence of hypothyroidism and hyperthyroidism separately using six studies. The pooled prevalence of hypothyroidism among COPD patients was 37% (n= 300) (95% CI: 14 to 60%), while the pooled prevalence of hyperthyroidism was 10% (n= 78) (95% CI: 3 to 17%). Overall, three studies were included to determine the association of thyroid dysfunction with the Global Initiative for Chronic Obstructive Lung Disease stage (GOLD); the results are presented in Figure 2. As shown in the figure, the odds of thyroid dysfunction were not significantly different among the severity levels of COPD (OR: 0.94, 95% CI: 0.70 to 1.28, p-value: 0.71). No significant heterogeneity was reported among the study results (I-square: 0%, p-value: 0.95).

Comparison of Characteristics Between Patients With and Without Thyroid Dysfunction

Table 3 presents a comparison of the characteristics between patients with and without thyroid dysfunction. Pooled analysis indicated that the mean age between the two groups was not significantly different. The number of males was also not significantly different between the two groups. Pulmonary function tests (PFT), including forced expiratory volume in one second (FEV1) and forced vital capacity (FVC), were likewise not significantly different between those with and without thyroid dysfunction. Finally, we compared the partial pressure of oxygen (PaO2) and partial pressure of carbon dioxide (PaCO2) between patients with and without thyroid dysfunction in COPD patients. As shown in Table 3, PaO2 and PaCO2 did not exhibit significant differences between the two groups (p-value > 0.05).

**Table 3:** Comparison of Characteristics Between Patients With and Without Thyroid Dysfunction MD: mean difference; CI: confidence interval; FEV1: forced expiratory volume in one second; FVC: forced vital capacity; PaO2: partial pressure of oxygen; PaCO2: partial pressure of carbon dioxide ^ Presented as odds ratio (95% CI) * Significant at p-value<0.05

Variable	MD (95 % CI)	P-value	I-square
Age (Years)	-0.98 (-4.46 to 2.49)	0.58	72%
Males ^	0.95 (0.44 to 2.05)	0.91	74%
FEVi (%)	1.51 (-3.69 to 6.72)	0.57	56%
FVC (%)	6.28 (-3.20 to 15.76)	0.19	16%
PaO2 (mmHg)	3.50 (-0.48 to 7.48)	0.08	46%
PaCO2 (mmHg)	1.93 (-2.74 to 6.61)	0.42	74%

Discussion

The objective of this meta-analysis was to ascertain the prevalence of thyroid dysfunction in individuals with COPD. The pooled prevalence of thyroid dysfunction among COPD patients was determined to be 45% (95% CI: 25% to 65%). To our knowledge, this is the first meta-analysis to assess the prevalence of thyroid dysfunction in COPD patients and to compare the characteristics of those with normal and abnormal thyroid function within the COPD population. Notably, hypothyroidism emerged as the most prevalent thyroid gland disorder in this context. Previous studies have consistently identified hypothyroidism as a common comorbidity in COPD patients. For instance, Prakash et al. conducted a study in 2014, analyzing 96 cases of acute COPD exacerbation, revealing that 64.58% of patients exhibited lower levels of T3, T4, and thyroid-stimulating hormone (TSH) [19]. Similarly, Singh et al. evaluated 201 cases of COPD, finding thyroid disorders in 64.6% of cases, with hypothyroidism diagnosed in 59.2% of cases and hyperthyroidism in 5.4% of cases [17].

The precise mechanism underlying thyroid dysfunction in COPD remains unclear, but it may be associated with hypoxemia and hypercapnia [20]. Karadag et al. observed that patients with COPD experiencing severe hypoxemia had lower levels of TSH and FT3 compared to those with milder hypoxia [9]. Conversely, some studies have suggested no correlation between thyroid hormones and PaO2 or PaCO2 [21-22]. In our present meta-analysis, we did not identify any significant relationship between PaO2 or PaCO2 and thyroid dysfunction. However, Terzano et al.'s study reported that PaO2 is significantly lower in patients with overt hypothyroidism and subclinical hypothyroidism compared to those with hyperthyroidism and without thyroid dysfunction [2]. Unfortunately, our meta-analysis could not conduct a subgroup analysis based on the type of thyroid dysfunction. Consequently, future studies should examine outcomes separately for different types of thyroid dysfunction.

Dimopoulou et al. [23] found that serum thyroid hormone levels were within the normal range in 46 stable COPD patients with varying degrees of disease severity. However, their study lacked a control group, and they did not observe any correlation between thyroid hormones and pulmonary function parameters in individuals with mild COPD. In our meta-analysis, we also observed no significant differences in pulmonary function tests, including FVC and FEV1, between individuals with and without thyroid dysfunction. Nevertheless, Dimopoulou et al. [23] identified a robust positive correlation between the TT3/TT4 ratio and PaO2 in patients with severe COPD, leading them to suggest that the severity of COPD might influence the peripheral metabolism of thyroid hormones. However, to affirm this finding, future studies with substantial sample sizes are required.

Despite age being a known risk factor for various health conditions, our meta-analysis suggests that age does not significantly influence the development of thyroid dysfunction in the COPD population. A previous study reported [24] that despite both COPD and thyroid dysfunction becoming more common with age, research has not found a strong association between the two in terms of development. While age plays a role in both conditions, other factors like smoking, inflammation, and medication use might have a stronger influence on thyroid dysfunction in COPD patients, regardless of age. Moreover, there are different types of thyroid dysfunction (hypothyroidism, hyperthyroidism), and their association with COPD and age might differ. Age might be more relevant for specific types [25].

Two of the studies incorporated into our analysis indicated that COPD patients with hypothyroidism experienced a higher frequency of exacerbations compared to those without this condition [6, 16]. The frequency of COPD exacerbations was positively correlated with TSH levels, and TSH value emerged as a significant determinant of exacerbation frequency [16]. Moreover, Bacakoğlu et al. demonstrated that low levels of fT3 and fT4 were associated with increased rates of invasive mechanical ventilation and mortality in patients experiencing respiratory failure [26]. Additional research is warranted to ascertain the impact of hypothyroidism on COPD exacerbations and to elucidate its effects on outcomes related to respiratory failure.

As a result, both functional and anatomical thyroid gland disorders are commonly found as comorbid conditions in individuals with COPD. Additionally, the ongoing inflammation and hypoxemia experienced by COPD patients have an impact on thyroid function [13]. Given that COPD manifests with symptoms such as shortness of breath, weakness, loss of appetite, and weight loss, the clinical indicators of hyperthyroidism and/or hypothyroidism may be obscured, posing challenges in diagnosis. Consequently, a delayed diagnosis of TGD can lead to adverse outcomes in COPD patients, as both hypothyroidism and hyperthyroidism negatively affect the respiratory system through distinct mechanisms [13]. A meticulous examination of the thyroid gland and the periodic measurement of thyroid function tests during the follow-up of COPD patients contribute to the early detection of TGD. The treatment of hypothyroidism associated with COPD follows the same approach as in patients without this comorbidity [24]. Although certain detrimental effects of hypothyroidism may be reversible with treatment, the impact of such treatment on lung functions and prognosis remains insufficiently understood, necessitating clarification through future studies.

The present meta-analysis possesses several limitations that warrant consideration. Firstly, a number of crucial characteristics were not consistently assessed across the majority of the included studies, potentially impacting the comprehensiveness of our analysis including FEV1, FVC, PaCO2, and Pa02. Secondly, due to the limited sample size available for subgroup analysis, we encountered constraints in conducting a detailed examination based on the specific type of thyroid dysfunction. This limitation may have hindered our ability to elucidate nuanced associations between thyroid dysfunction and COPD outcomes, potentially limiting the generalizability and depth of our findings. Furthermore, it is noteworthy that seven out of the nine studies incorporated into our analysis were of a cross-sectional nature, implying inherent limitations in establishing causation or assessing the temporal relationship between thyroid dysfunction and COPD. In light of these limitations, there arises a need for prospective studies that can offer valuable insights into the dynamic interplay between thyroid dysfunction and COPD. Such studies could explore the potential effects of thyroid dysfunction on COPD progression, its impact on the duration of hospital stay, and its association with mortality and morbidity.

Recommendations

Future research should prioritize larger and more diverse cohorts to overcome sample size limitations, enabling detailed subgroup analyses of specific thyroid dysfunctions in COPD. Longitudinal and mechanistic studies are crucial for elucidating temporal associations and underlying pathways, shedding light on the complex interplay between thyroid function and COPD progression. Additionally, interventional trials and subgroup analyses based on patient characteristics can inform targeted management strategies for improved COPD outcomes, enhancing patient care and quality of life.

## Conclusions

In conclusion, the meta-analysis revealed a pooled prevalence of 45% for thyroid dysfunction in COPD patients, with hypothyroidism being the most common thyroid gland dysfunction. The study identified a lack of significant associations between thyroid dysfunction and COPD severity or various characteristics, highlighting the need for further research, particularly with larger sample sizes and prospective designs. Additionally, the impact of thyroid dysfunction on COPD exacerbations and respiratory failure outcomes requires more in-depth exploration. Despite certain limitations, this analysis underscores the importance of vigilant thyroid assessment in COPD patients for timely detection and management.
